# Novel Phenotypic Outcomes Identified for a Public Collection of Approved Drugs from a Publicly Accessible Panel of Assays

**DOI:** 10.1371/journal.pone.0130796

**Published:** 2015-07-15

**Authors:** Jonathan A. Lee, Paul Shinn, Susan Jaken, Sarah Oliver, Francis S. Willard, Steven Heidler, Robert B. Peery, Jennifer Oler, Shaoyou Chu, Noel Southall, Thomas S. Dexheimer, Jeffrey Smallwood, Ruili Huang, Rajarshi Guha, Ajit Jadhav, Karen Cox, Christopher P. Austin, Anton Simeonov, G. Sitta Sittampalam, Saba Husain, Natalie Franklin, David J. Wild, Jeremy J. Yang, Jeffrey J. Sutherland, Craig J. Thomas

**Affiliations:** 1 Lilly Research Laboratories, Eli Lilly and Company, Indianapolis, Indiana, United States of America; 2 Division of Preclinical Innovation, National Center for Advancing Translational Sciences, National Institutes of Health, Bethesda, Maryland, United States of America; 3 Indiana University School of Informatics and Computing, Bloomington, Indiana, United States of America; Broad Institute of Harvard and MIT, UNITED STATES

## Abstract

Phenotypic assays have a proven track record for generating leads that become first-in-class therapies. Whole cell assays that inform on a phenotype or mechanism also possess great potential in drug repositioning studies by illuminating new activities for the existing pharmacopeia. The National Center for Advancing Translational Sciences (NCATS) pharmaceutical collection (NPC) is the largest reported collection of approved small molecule therapeutics that is available for screening in a high-throughput setting. Via a wide-ranging collaborative effort, this library was analyzed in the Open Innovation Drug Discovery (OIDD) phenotypic assay modules publicly offered by Lilly. The results of these tests are publically available online at www.ncats.nih.gov/expertise/preclinical/pd2 and via the PubChem Database (https://pubchem.ncbi.nlm.nih.gov/) (AID 1117321). Phenotypic outcomes for numerous drugs were confirmed, including sulfonylureas as insulin secretagogues and the anti-angiogenesis actions of multikinase inhibitors sorafenib, axitinib and pazopanib. Several novel outcomes were also noted including the Wnt potentiating activities of rotenone and the antifolate class of drugs, and the anti-angiogenic activity of cetaben.

## Introduction

Lead-generation screening strategies in drug discovery are often categorized as either target-based or phenotypic and these descriptors remain relevant today [1]. As the molecular biology and genomics revolutions provided invaluable insight into cellular targets and signaling pathways, a trend toward target-based approaches began to dominate many drug discovery programs. These efforts have yielded an impressive collection of small molecule tools with highly defined primary pharmacologies, including several approved therapeutics [2, 3]. However, a recent analysis of drugs approved between 1999 and 2008 demonstrated that phenotypic screening approaches represented the most successful method for discovering first-in-class drugs [1, 4]. Reflecting these trends, phenotypic screening has witnessed a resurgence in popularity in lead-generation screening efforts.

Drug repositioning has also undergone a renaissance in recent years with interest from both academia and industry [5]. Approved drugs for diabetes (metformin) are being explored in cancer therapy, the precarious sedative thalidomide is an effective treatment for erythema nodosum leprosum (leprosy) and the approved hormone therapy tamoxifen is being examined in bipolar disorder. The path to repositioning of these drugs (and others) was undoubtedly facilitated by the limited need to repeat costly and time-consuming preclinical studies and phase I safety trials for use of these already-approved agents. The justifications for many repositioning efforts are rooted in mechanistic insight (for instance, targeting of protein kinase C by tamoxifen) or clinical observation, and the majority of drug repositioning efforts remain iterative in nature. Unfortunately, we remain woefully unaware of the full mechanistic, and therefore therapeutic, potential for the vast majority of approved drugs. Undoubtedly, individual examination of specific medicines will continue to highlight novel uses for approved drugs *via* the progressive and deliberate explorations of researchers in search of new therapies. However, akin to the renewed interest in phenotypic screening for lead-generation, there exists an intriguing potential for the examination of large drug libraries in phenotypic- and mechanism- informing whole-cell assays to detect novel phenotypes associated with approved therapies.

The screening of small molecule drug collections is becoming more commonplace and multiple small molecule vendors now offer drug library collections for this purpose [6]. While these collections are incredibly useful, building an exhaustive collection of all regulatory-agency-approved, obtainable, and HTS-suitable small molecule drugs for screening is a daunting endeavor. The National Center for Advancing Translational Sciences (NCATS) has compiled the largest public repository of approved and clinical phase drugs [the NCATS Pharmaceutical Collection (NPC)] and is regularly applying this collection in repositioning studies, toxicological assessments, and for chemical genomic profiling [7]. The details of this collection are freely available (www.ncats.nih.gov/expertise/preclinical/pd2) and results from specific screens are provided at www.ncats.nih.gov/expertise/preclinical/pd2 and through the PubChem database (https://pubchem.ncbi.nlm.nih.gov/)(AID 1117321). To date, this collection has been utilized to define potential new therapies for chronic lymphocytic leukemia (CLL) and malaria [8, 9].

Evaluation of the NPC library in highly specified phenotypic assays continues to yield intriguing results. Importantly, public release of all data is intended to ensure that screening results can be evaluated by the scientific community where insight into compelling results may lead more rapidly to translation. The Open Innovation Drug Discovery (OIDD) program at Lilly (https://openinnovation.lilly.com/dd/) uses a collaborative approach intended to guide discovery and translation through partnership [10]. The OIDD screening panel offers unique and well vetted assessment of small molecules in *in vitro* models of disease. At the close of 2014, the OIDD program had screened more than 35,000 small molecules from over 400 institutions worldwide; several novel chemotypes had been advanced to preclinical models to assess their appropriateness for further examination. To leverage both the scope of the NPC and the availability of phenotypic disease models included in the OIDD panel, NCATS and Lilly established a broad collaboration to profile the NPC in selected OIDD phenotypic modules. At the time that the work was conducted, these included Wnt potentiation (osteoporosis model), Insulin and GLP-1 secretion (diabetes models), KRAS-Wnt synthetic lethality, and Angiogenesis (cancer models). This communication provides a summary of the results obtained from the characterization of 2460 clinical phase/approved drugs in the five OIDD phenotypic modules listed above. Importantly, the results of these studies are publicly available online at www.ncats.nih.gov/expertise/preclinical/pd2 via the Pubchem Database (https://pubchem.ncbi.nlm.nih.gov/)(AID 1117321).

## Materials and Methods

### NPC library

The compilation of molecular entities that are considered drugs has been reported previously and NCATS maintains an online database that allows researchers free access to this non-redundant list and the methods by which it was compiled (www.ncats.nih.gov/expertise/preclinical/pd2)[7]. In total, 2,509 active pharmaceutical ingredients were evaluated in five selected OIDD phenotypic screening modules. The full listing of drugs tested in this study is provided via online at www.ncats.nih.gov/expertise/preclinical/pd2 and via the PubChem Database (https://pubchem.ncbi.nlm.nih.gov/)(AID 1117321).

### OIDD Phenotypic Assay Modules

Approved and experimental drugs included in NPC were tested in at least two concentrations in single point format. Compound activity was confirmed by generating 10-point concentration response curves (CRC). Where noted, verified actives from the primary assay were subsequently tested in confirmatory assays in 10-point concentration response to better define the overall biological phenotypes.

### Wnt, insulin secretion and angiogenesis

The methods for the Wnt activation, insulin secretion, and angiogenesis models have been previously reported including details on reagents, cell lines, methods for data collection, methods for data examination and analysis of assay robustness [10]. Briefly, the Wnt pathway potentiation module monitors β-catenin translocation and alkaline phosphatase (ALP) activity in the multilineage potential C2C12 cell line in the presence of an EC_20_ of Wnt3A conditioned media. The insulin secretion assay utilizes a homogeneous immunoassay to quantify insulin secretion from the INS-1E cell line in either high glucose (5 mM) or low glucose (0.1 mM) conditions. A thallium flux assay to quantify the inhibition of tonic K^+^ channel activity under 0.1 mM glucose conditions was also performed in INS-1E cells. This was essentially developed and optimized using the FluxOR system (Life Technologies). The angiogenesis assay module measures endothelial tube formation and cell nuclei number within co-cultures of human clonal endothelial colony forming cells (ECFCs) and adipose-derived stem cells (ADSCs) treated with VEGF. Standard controls were utilized for each assay module including the approved drug glibenclamide (for the insulin secretion assay module), a GSK3β inhibitor (for the Wnt activation module) and the approved VEGFR inhibitor Sutent (for the angiogenesis module).

### KRAS/Wnt synthetic lethal

The KRAS synthetic lethal module seeks to identify compounds that are selectively cytotoxic to cell lines bearing KRAS mutation vs. wild type under conditions that mimic tumor metastasis using non-adherent and non-proliferating cells. A panel of 7 colon cancer cell lines with various combinations of KRAS, APC, PI3K and BRAF mutations were selected for identifying relationships between genotypes and compound sensitivity. To determine the effect of compounds on viability, cells (6–10K/well) were plated in 384 well Ultra Low Attachment plates (Corning #3827) in RPMI 1640 containing 2 mM L-glutamine (Gibco #11875) and supplemented with 25 mM HEPES. Compounds were diluted in DMSO and 100 nL added to wells using a Pintool head (V&P Scientific) on a Beckman Multimek liquid handler (0.25% DMSO final concentration). After 72h, cell number was estimated using Cell TiterGlo (Promega #G8462). In the primary screening assay, 3 cell lines with activating KRAS mutations (SW480, DLD-1 and HCT116) were assayed at 0.2, 2.0 and 20 μM of test compounds. Z’ values [11] range from 0.49 to 0.76 with a mean of 0.59. Compounds producing >70% inhibition @ 20 μM and >40% inhibition at 2 μM were selected for generating concentration response curves across the full 7 cell line panel. The positive control for all assays was staurosporine (10 μM).

To identify compounds that influence the interactions between Wnt and MAPK pathways that are relevant to colorectal cancer pathology, an additional assay with HCT116 cells treated with a GSK3β inhibitor, which activates the Wnt pathway, was also included. For these assays the GSK3β inhibitor LY2090314 (20 nM) was added to cells just prior to plating and compound addition. Compound additions and viability assays were processed as described above.

### GLP-1 secretion

Human NCI-H716 cells (CCL-251, ATCC) were propagated in suspension at 37°C, in a humidified incubator at 5% CO_2_ in RPMI 1640 medium (Invitrogen) supplemented with 10% FBS (US Certified heat inactivated, Invitrogen), 2 mM L-Gln (Invitrogen), 10 mM HEPES (Hyclone) and Anti Biotic/Anti Mycotic (Hyclone). The cells were grown overnight in media comprised of DMEM (SH30284.01, Hyclone), 10 mM HEPES, 10% FBS, and Anti Biotic/Anti Mycotic. On the day of the assay the cells were washed twice in HBSS plus 0.1% (w/v) BSA plus 1% (w/v) DPP-IV Inhibitor and re-suspended in HBSS (+ BSA, + DPP-IV) and plated at 10,000 cells per well on poly-D-lysine 384 well plates black with clear bottom (Greiner or BD). Cells were dosed with compound starting at a concentration of 40 μΜ with a 3 fold serial dilution for a dose response. Cells were treated with compound for 2 hours at 37°C under ambient conditions. The mouse enteroendocrine-like STC-1 cell line [12] was used analogously for GLP-1 secretion experiments with the exception that the cells were cultured in the DMEM containing media and plated at 12,000 cells/well and DPP-IV inhibitor was not used. Secreted GLP-1 in supernatants was quantified using an in house constructed homogenous AlphaLISA assay in a 384-well format. Samples were read on an Envision (Perkin Elmer) and the assay was calibrated to synthetic GLP-1 peptides (Bachem, Torrance, CA). For single point and concentration response activity the % stimulation is calculated based on normalizing the GLP-1 secreted to compounds which produce a robust and close to maximal response of GLP-1 secretion. For NCI cells an internally discovered compound was used due to the lack of suitable reference standards in this biological system. In the STC-1 cells the GPR43 allosteric agonist (2S)-2-(4-chlorophenyl)-3-methyl-N-(1,3-thiazol-2-yl)butanamide) was utilized [13]. Both GLP-1 secretion assays performed robustly with Z’ = 0.66 (NCI, n = 27) and Z’ = 0.49 (STC, n = 31) respectively.

### HeLa G2/M Assay

The G2/M assay module was not used for screening the NPC collection. It was used in this work to profile actives from the other OIDD phenotypic modules because of its utility in understanding mechanism. Specifically, activity in the angiogenesis or Wnt modules frequently co-occurs with activity in the G2/M module; a compound which causes cell cycle arrest may be of lower interest as an anti-angiogenic or Wnt pathway potentiator, because effects on those pathways are likely to be secondary.

### Secondary Kinase Assays

To investigate their mechanism of action, confirmed actives from the Angiogenesis and Wnt modules were profiled at 3 concentrations (0.2, 2.0 and 20 μM) in through-plate mode using the CerepLANCE kinase assays (http://www.cerep.fr/cerep/users/pages/productsservices/kinasePlatform.asp). The Entrez gene symbols for the tested kinases are FLT3, GSK3β, JAK2, KDR and RPS6KB1). The 3 single concentration estimates are converted to an IC_50_ using constrained logistic fits [14].

### Identification of active compounds

Compounds were tested through a hierarchal flow scheme composed of 35 assays in a stepwise manner (S1 Table). Initially, all compounds were tested in two or three concentrations (screening assays), and those with activity exceeding a defined cutoff were retested in the screening assay in full concentration response to confirm activity. Confirmatory assays are used to investigate down-stream effects consistent with the primary activity readout and/or translation to other systems. Finally, profiling assays are used to investigate mechanism of action.

For the angiogenesis module, actives from the screen (assay 1) are defined as those with % inhibition ≥ 40 (i.e. a decrease in tube area) in either of two tested concentrations (2 and 10 μM). The actives were advanced to the primary assays (assays 2 and 3) which yield the IC_50_ of compounds for inhibiting tube area and nuclear count, measures of vasculogenesis and overt cytotoxicity, respectively. Actives are defined as those compounds with an IC_50_ ≤ 5 μM for inhibiting tube area, and a selectivity ratio > 10: the potency of inhibiting cell viability (undesired) occurs at a 10x or greater concentration that the effect on tube area (desired). We also retain compounds where the selectivity ratio is qualified and greater than 2 (e.g. tube area IC_50_ = 5 μM and nuclear area IC_50_ > 10 μM results in a selectivity ratio of >2). Compounds meeting these criteria were tested in the G2/M profiling assays (14–18) and kinase panel (assays 31–35) to investigate mechanisms of anti-angiogenic activity.

For the Wnt potentiation module, actives from the screen (assay 4) are defined as those with % stimulation ≥ 40 (i.e. increased nuclear β-catenin) in either of two tested concentrations (2 and 10 μM) in C2C12 cells. The actives were confirmed in concentration response using the primary assay (assay 5) which measures the EC_50_ of the effect on β-catenin accumulation, and an additional assay measuring increases in cellular ALP activity (assay 6), a down-stream effect of Wnt activation. Compounds active in the primary assays (EC_50_ ≤ 5 μM) were tested in the kinase panel and G2/M profiling assays.

Compounds were screened in the insulin secretion assay (assay 7) at concentrations of 2 and 10 μM in the presence of 5 mM glucose using INS-1E cells. Compounds with ≥ 20% stimulation were advanced to concentration response testing using assay 8 for EC_50_ determination; compounds yielding an EC_50_ ≤ 5 μM were tested in concentration response at low glucose (0.1 mM; assay 9) to assess their selectivity for increasing insulin secretion at high glucose levels; compounds were then tested in a phenotypic K^+^ flux assay at 0.1 mM glucose to identify putative modulators of ATP-dependent K^+^ flux in INS-1E cells (assay 10). Actives are defined as those with EC_50_ ≤ 5 μM at 5 mM glucose and a corresponding low-glucose EC_50_ at least 10 fold higher. The K^+^ flux assay is used to understand the mechanism of action of active compounds.

The GLP-1 screen was used to identify compounds with % stimulation ≥ 20 at concentrations of 2 or 10 μM in NCI-H716 cells (assay 11). Screen actives were followed up in concentration response using NCI-H716 cells (assay 12) and mouse STC-1 cells (assay 13). Compounds having GLP-1 secretion EC_50_ ≤ 5 μM in either assay are defined as actives.

The KRAS/Wnt synthetic lethal module encompasses 5 sub-modules. Initially, compounds were screened at 0.2, 2 and 20 μM in the DLD-1, SW480, HCT116 and GSK3B inhibitor pre-treated HCT116 assays (assays 19–22). Constrained logistic fits were formed to yield approximate IC_50_s [14], and compounds with IC_50_ less than 2 μM were retested in 10 point concentration response curves in the same lines (assays 23–26), in addition to Colo320, HT-29, RKO and SNU-C1 cells (assays 27–30); the module was removed from the OIDD panel part way through this project and not all screen actives were retested in 10-point curves. Because these lines harbor different mutations, the dependence of drug response on various mutations was assessed by comparing the IC_50_ of lines with mutation vs. those without, and quantified by calculating selectivity ratios. For the KRAS sub-module, the most potent IC_50_ across KRAS-mutant lines (HCT116, DLD-1 and SW480) was compared to the most potent IC_50_ against the wild type lines Colo320 and SNU-C1; compounds with ≥ 10x greater potency in KRAS-mutant lines were defined as actives. A similar approach was used for the BRAF sub-module, where compounds having ≥ 10x greater potency in the BRAF-mutant lines RKO and HT-29 than the wild type lines HT-29, DLD-1, HCT116, SW480 and SNU-C1 were defined as actives. Actives in the Colo320-resistance module were identified by comparing the IC_50_ in Colo320 cells to the geometric mean of IC_50_s for the other 6 cell lines, by selecting those with ≥ 10x lower potency in Colo320. Two additional modules were included for the purpose of identifying potential WNT pathway modulators. HCT116 cells were treated with a GSK3β inhibitor, which stimulates WNT pathway signaling, and compound activities in basal vs treated cells were compared. Compounds with ≥ 10x greater potency in the untreated cells (Basal HCT116 sub-module), or those with ≥ 10x greater potency in the GSK3β pre-treated cells (Wnt-stim HCT116 sub-module) are potential Wnt pathway modulators.

All results are provided with qualifier and value. By definition, all single concentration results have qualifier “=“. For IC_50_ values, these have qualifier ‘ = ‘ when the IC_50_ falls within the range of studied concentrations, and have qualifier “>” otherwise (i.e. no response in range of studied concentrations). Where replicate results are available for a given compound and assay, we apply the following procedure to summarize the replicates, and use the summarized value for further analyses. For single concentration testing, we average results obtained at the same concentration. Where multiple IC_50_ results are available, and at least one or more have qualifier ‘ = ‘, we compute the geometric mean of all results with the ‘ = ‘ qualifier and discard those (if any) with qualifier “>”. Finally, if all results have qualifier “>”, we retain the one with the largest value (e.g. IC_50_ >10 μM and IC_50_ >20 μM is represented with the single value >20 μM).

### Statistical Analysis

All assays were validated in accordance with the published Lilly-NIH Chemical Genomics Center guidelines for assay enablement and statistical validation (http://www.ncgc.nih.gov/guidance/index.html).

## Results and Discussion

### Summary of screening results

A total of 2,509 small molecules representing the active pharmaceutical ingredient of an approved or developmental drug were screened in five OIDD phenotypic assay modules. A total of 60,473 screening tests (single concentration testing) and 5,471 concentration-response experiments were performed. The percent actives for each assay module ranged between 0.6% and 3.7% of the library (Table 1). Interestingly, the overall activity rate of OIDD phenotypic assays obtained by screening the NPC collection are similar to those obtained by screening compounds submitted by the full community of OIDD participants. This is noteworthy, as the latter are small molecules of unknown function originating from academic, biotech, and research institute laboratories that are structurally distinct from known drugs and compounds in the Lilly corporate collection [10, 15]. As the use of privileged, highly annotated and focused screening libraries becomes more commonplace it will be important to compare their hit rates with those of unbiased screening libraries to see if this commonality persists.

**Table 1:** Comparing OIDD phenotypic module hit rates obtained by screening NPC and OIDD compound collections.

Module	NPC hit rate ^a^	OIDD hit rate^b^
Angiogenesis	3.7% (92)	5.1% (866)
Wnt potentiation	2.4% (61)	1.7% (296)
Insulin secretion	0.6% (15)	0.3% (46)
GLP-1 secretion	0.7% (17)	0.4% (71)
KRAS	1.4% (35)	1.0% (174)

^a^ the hit rate is the percent of 2,509 compounds screened having IC_50_ ≤ 5 μM in the primary concentration-response assays and meeting the selectivity criteria described in methods (e.g. reduction in tube area without cytotoxicity in the angiogenesis module).

^b^ hit rate from screening 17,031 OIDD compounds as described in ref [10].

### Identification of active compounds

**Fig 1:**
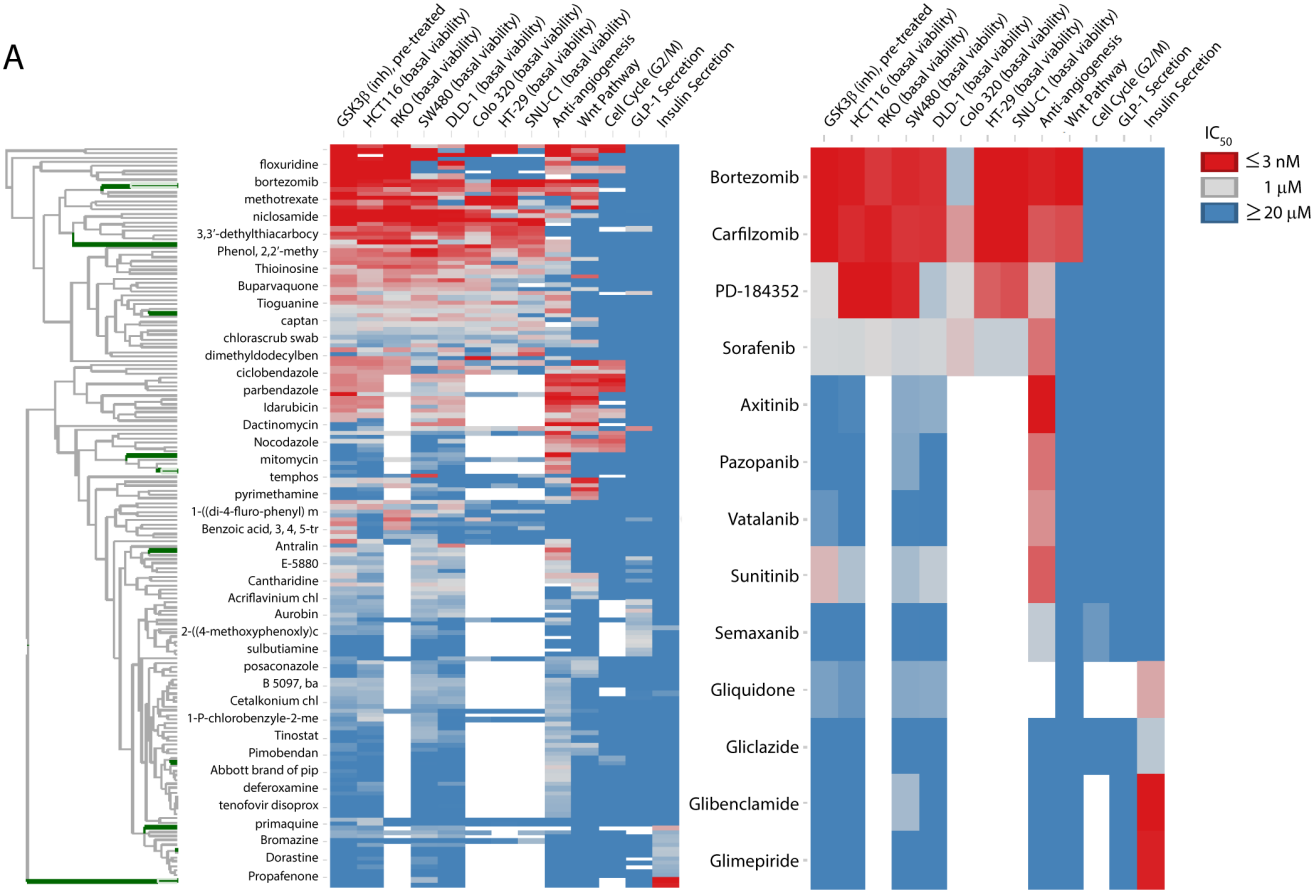
Heatmap representation of activity results for 173 active compounds vs. primary assays from 6 OIDD phenotypic assay modules. The clustering was performed on log10-transformed IC_50_s from S2 Table. The color scale ranges from blue (IC_50_ ≥ 20 uM) to red (IC_50_ ≤ 3 nM). The positions of compounds shown in the inset are highlighted in green on the compound dendrogram.

Utilizing the criteria defined for the primary assays (methods), 173 molecules with activity in one or more modules were identified (Table 2; S2 Table). Subsequent testing of confirmed primary screen actives in secondary assays and across phenotypic assay modules can provide important information concerning compound promiscuity, desired/undesired phenotypes, and potential mechanisms of action. While the majority of the active compounds (123 / 173 or 71% percent) are active in only one module, hierarchical clustering highlights a continuum of compound promiscuity across these results (Fig 1).

**Table 2:** IC_50_ / EC_50_ in μM for selected compounds across phenotypic assay modules.

Module^a^	Compound	Anti-Angio	G2/M	GLP1sec.	Ins.sec.	Wntpot.	Colo320 viab.	DLD-1 viab.	HCT-116viab.	HT-29 viab.	RKO viab.	SNU-C1 viab.	SW480 viab.	HCT-116 (GSK3β) viab.
Wnt	methotrexate	20^b^	20	20.1	20.1	**0.012**	0.001	0.70	0.001	0.001	0.001	20	0.001	0.001
	pyrimethamine	20.1^c^	20	20.1	20.1	**0.068**	NA	20	18.2	NA	NA	NA	12.5	20
	nolatrexed	7.4	20	20.1	20.1	**0.24**	20	20	3.5	4.7	0.34	20	2.2	0.83
	trimetrexate	4.4	20	20.1	20.1	**0.003**	0.39	0.20	0.023	0.021	0.003	5.1	0.059	0.004
	raltitrexed	0.35	20	20.1	20.1	**0.004**	20	20	0.004	20	0.003	20	0.001	0.002
	metoprine	20.1	20	20.1	20.1	**0.030**	2.0	0.63	0.41	0.93	0.12	2.2	0.51	0.059
	dasatinib	20	20	20.1	20.1	**0.007**	NA	3.5	11.9	NA	NA	NA	7.0	20
	rotenone	0.026	0.31	20.1	20.1	**0.25**	NA	0.35	1.2	NA	NA	NA	0.16	0.067
Insulin	glibenclamide	20.1	NA	20.1	**0.002**	20.1	NA	20	20	NA	NA	NA	2.9	20
	glimepiride	20.1	NA	20.1	**0.004**	20.1	NA	20	20	NA	NA	NA	20	20
	gliquidone	NA	NA	NA	**0.23**	20.1	NA	5.1	10.1	NA	NA	NA	5.5	7.0
	gliclazide	20.1	20	20.1	**1.8**	20.1	NA	20	20	NA	NA	NA	20	20
	repaglinide	20.1	20.1	20.1	**0.030**	20.1	NA	20	20	NA	NA	NA	20	20
	amitriptyline	20.1	20	20.1	**3.0**	20.1	NA	20	20	NA	NA	NA	10.1	20
	butriptyline	20.1	20	20.1	**3.6**	20.1	NA	8.1	6.2	NA	NA	NA	7.8	20
	metitepine	20	20	20.1	**2.9**	20	NA	4.4	3.8	NA	NA	NA	1.9	4.6
	propafenone	20.1	20	20.1	**4.9**	20.1	NA	20	20	NA	NA	NA	20	20
	etafenone	20.1	20	NA	**3.9**	20.1	NA	20	11.2	NA	NA	NA	16.9	20
	mebeverine	20.1	20	20.1	**2.0**	20.1	NA	20	20	NA	NA	NA	20	20
Angio	semaxanib	**1.6**	9.1	20.1	20.1	20.1	NA	20	20	NA	NA	NA	19.7	20
	vatalanib	**0.11**	20	20.1	20.1	20.1	NA	20	20	NA	NA	NA	19.5	10.5
	axitinib	**0.001**	20	20.1	20.1	20.1	NA	4.5	16.8	NA	NA	NA	5.1	20
	pazopanib	**0.047**	20	20.1	20.1	20.1	NA	20	20	NA	NA	NA	5.7	20
	sunitinib	**0.025**	20	20.1	20	20.1	NA	1.6	2.3	NA	NA	NA	2.8	0.36
	cetaben sodium	**0.37**	20	20.1	20.1	20.1	NA	10.6	20	NA	NA	NA	4.4	7.4
KRAS	PD-184352	0.37	20	20.1	20.1	20.1	**0.85**	**2.2**	**0.002**	**0.026**	**0.003**	**0.017**	**0.005**	**0.97**
	bortezomib	0.004	20	20.1	20.1	0.001	**2.8**	**0.006**	**0.004**	**0.003**	**0.008**	**0.003**	**0.005**	**0.002**
	carfilzomib	0.020	20	20.1	20.1	0.015	**0.14**	**0.007**	**0.006**	**0.003**	**0.004**	**0.001**	**0.008**	**0.002**
	simvastatin	1.5	20	3.3	20.1	20.1	**4.9**	**20**	**20**	**20**	**20**	**20**	**5.9**	**4.7**
	cerivastatin	0.089	20	20.1	20.1	19.9	**1.9**	**2.5**	**0.24**	**1.2**	**1**	**1.9**	**0.14**	**0.053**
	fluvastatin	0.98	20	20.1	20.1	20.1	**13.0**	**20**	**0.97**	**20**	**19.2**	**20**	**1.7**	**0.040**

^a^ the module in which compound exhibits activity; only the IC_50_ value in the primary assay (or most potent of multiple primary assays) is shown; all compounds meet the selectivity criteria described in the methods.

^b^ values shown as 20 indicate that the compound was tested in concentration-response and showed no response at the highest concentration tested (e.g. IC50 > 20 μM).

^c^ values shown as 20.1 indicate that compound was inactive in single concentration testing and not advanced to concentration-response profiling. Refer to S2 Table for the full list of 173 actives and S4 Table and S5 Table for the complete results from concentration-response and single-concentration testing, respectively.

The insulin secretion screen identified 16 active compounds (0.6% hit rate); among them are several sulfonylureas used to treat type II diabetes: glibenclamide (EC_50_ = 2.5 nM), glimepiride (4.1 nM), gliquidone (230 nM) and gliclazide (1.8 μM). Also identified was repaglinide (27 nM), an approved insulin secreting agent that acts at the same biological target (SUR1/Kir6.2) as the sulfonylureas [16]. Repaglinide and glibenclamide both stimulated insulin secretion at low glucose concentrations. The purpose of the 0.1 mM glucose assay is to eliminate definitive glucose-independent insulin secretagogues from further study. All sulfonylureas and metaglinides are mechanistically glucose-independent; however, this cannot always be demonstrated outside of pancreatic beta cell. With the exception of efavirenz which also exhibited activity in the GLP-1 secretion assay (and is therefore likely a general secretagogue), the insulin secretagogues exhibited no activity in the other modules. While their insulin secretion potencies are modest (EC_50_ values between 2 and 5 μM), CNS agents are prevalent among the remaining actives. There were three chemotypes identified as actives: tricyclic antidepressants (metitepine, amitriptyline, butriptyline), phenylpropanone anti-arrhythmics, and the muscarinic antagonist mebeverine. The effects of tricyclics on insulin levels have been studied in rats [17] and humans [18]. Moreover, it is well described that various monoaminergic GPCRs regulate beta cell function, including muscarinic, dopaminergic, and adrenergic receptors and these effects can be observed in INS-1 cells [19–21]. However, it must be noted that clinically used tricyclic drugs have an extensive polypharmacology, typically with nanomolar affinities for numerous monoaminergic transporters and GPCRs. Examining the activities of amitriptyline (IC_50_ = 6.2 μM) and butriptyline (>20 μM) in the K^+^ channel assay, it is clear that inhibition of K^+^ channel activity is putative mechanism of action of these compounds (metitepine was not tested). Similarly, the insulin secretion activity of the sodium channel blockers etafenone (K^+^ assay IC_50_ = 6.7 μM) and propafenone (5.4 μM) likely also arises from activity at potassium channels. In line with this, a recent report demonstrates that propafenone has micromolar potencies for the inhibition of a number of K^+^ channels including SUR2/Kir6.2 (IC_50_ = 2.2 μM) as measured by electrophysiology [22]. Analogously, mebeverine, an antimuscarinic approved for use in the treatment of irritable bowel syndrome, was equipotent in the insulin secretion (EC_50_ = 2.0 μM) and K^+^ channel (IC_50_ = 1.7 μM) assays indicating that activity on the pancreatic beta-cell KATP/sulfonylurea receptor complex (SUR1/Kir6.2) is a probable mechanism of action. In summary, the insulin secretion module uncovered several novel compound activities, however the limited mechanistic data we have suggest this is due to modulation, either directly or indirectly, of a known and well drugged target: the pancreatic beta cell ATP-dependent potassium channel complex (SUR1/Kir6.2). Due to the biological function of SUR1/Kir6.2 it is likely that the identified compounds would be glucose-independent *in vivo* and therefore have a propensity for hypoglycemia. One would expect such effects to be readily observed in clinical trials. Thus it is likely that these compounds do not translate to the human state or that insufficient exposure is achieved to induce these effects in humans.

The GLP-1 secretion module identified 17 actives (0.7% hit rate), mostly in the 1–5 μM range. This group of compounds had an unusually broad range of activities in the phenotypic modules. Upon examination of active compound structures it is clear that the GLP-1 assay module has a propensity to uncover a variety of idiosyncratic cytotoxic and likely false positive compounds. All but 4 compounds tested inhibited cell viability in the KRAS module cell lines. Antibiotics, antiseptics, antifungals, and antihelminthics are prevalent among the actives. Notably, potent actives in both STC-1 and NCI-H716 cell assays included germanium and mercury containing compounds (nitromersol, propagermanium). Four compounds had interesting phenotypes. The first, hydroquinone, a reactive and very low molecular weight antiseptic, has low probability of being a useful tool compound. Similarly, the 193 Da molecule 2-mercapto-5-(trifluoromethyl)anilinium chloride contains a free thiol and would be highly reactive *in vitro* and *in vivo*. The synthetic vitamin B1 analogue sulbutiamine is used for the treatment of asthenia with an unclear mode of action, although it is postulated that neuronal thiamine supplementation is involved in mediating efficacy [23]. Lastly, erythrosin B is an FDA approved food dye known as Red number 3 and a known photosensitizer and well-described promiscuous inhibitor [24]. In our view, the compounds identified in the GLP-1 secretion screen lack human therapeutic potential or utility as tool compounds.

We identified 92 actives from the angiogenesis module (3.7% hit rate); these compounds inhibit endothelial tube formation without significantly affecting viability of the co-cultured ECFC/ADSC. All but 15 compounds have similar potency (i.e. within 10-fold) in the G2/M or KRAS modules, underscoring the prominence of cytotoxicity and/or cellular energetics as common mechanisms underlying anti-angiogenic activity. This is consistent with the prevalence of anti-bacterial, anti-protozoal, anti-helminthic, and anti-neoplastic compounds present among the actives, and underscores the importance of utilizing additional cell health assays beyond monitoring the viability of the ECFC/ADSC cells in the primary assay. Compounds selectively active for inhibition of angiogenesis includes the known receptor-tyrosine kinase (RTK) inhibitors axitinib (IC_50_ = 1.3 nM), sunitinib (25 nM), pazopanib (47 nM), vatalanib (110 nM) and semaxanib (1.6 μM).

The Wnt module screen identified 61 active compounds (2.4% hit rate). As observed for the angiogenesis actives, most actives are cytotoxic compounds with activity in the G2/M and KRAS modules. Because the assay measures increased nuclear β-catenin on a per-cell basis, the prevalence of cytotoxic compounds among the actives is consistent with the known role of Wnt signaling in cell survival. Among the Wnt actives with higher selectivity are dasatinib (IC_50_ = 6.7 nM) and a subset of anti-folates (pyrimethamine, metoprine and nolatrexed; Wnt IC_50_s between 30 and 240 nM).

The KRAS/Wnt synthetic lethal module was designed to identify compounds with greater anti-proliferative potency in colorectal cancer cell lines harboring KRAS, BRAF and/or Wnt activating mutations. IC_50_ values from 8 primary assays were combined in various ratios to identify compounds with preferential effects in a subset of cell lines (methods). Across the 5 sub-modules, 35 actives were identified (1.4% hit rate). Most actives were known anti-bacterial, anti-protozoal, anti-helminthic and anti-neoplastic agents. Noteworthy examples include the KRAS-dependence of several statins and anti-folates.

**Fig 2:**
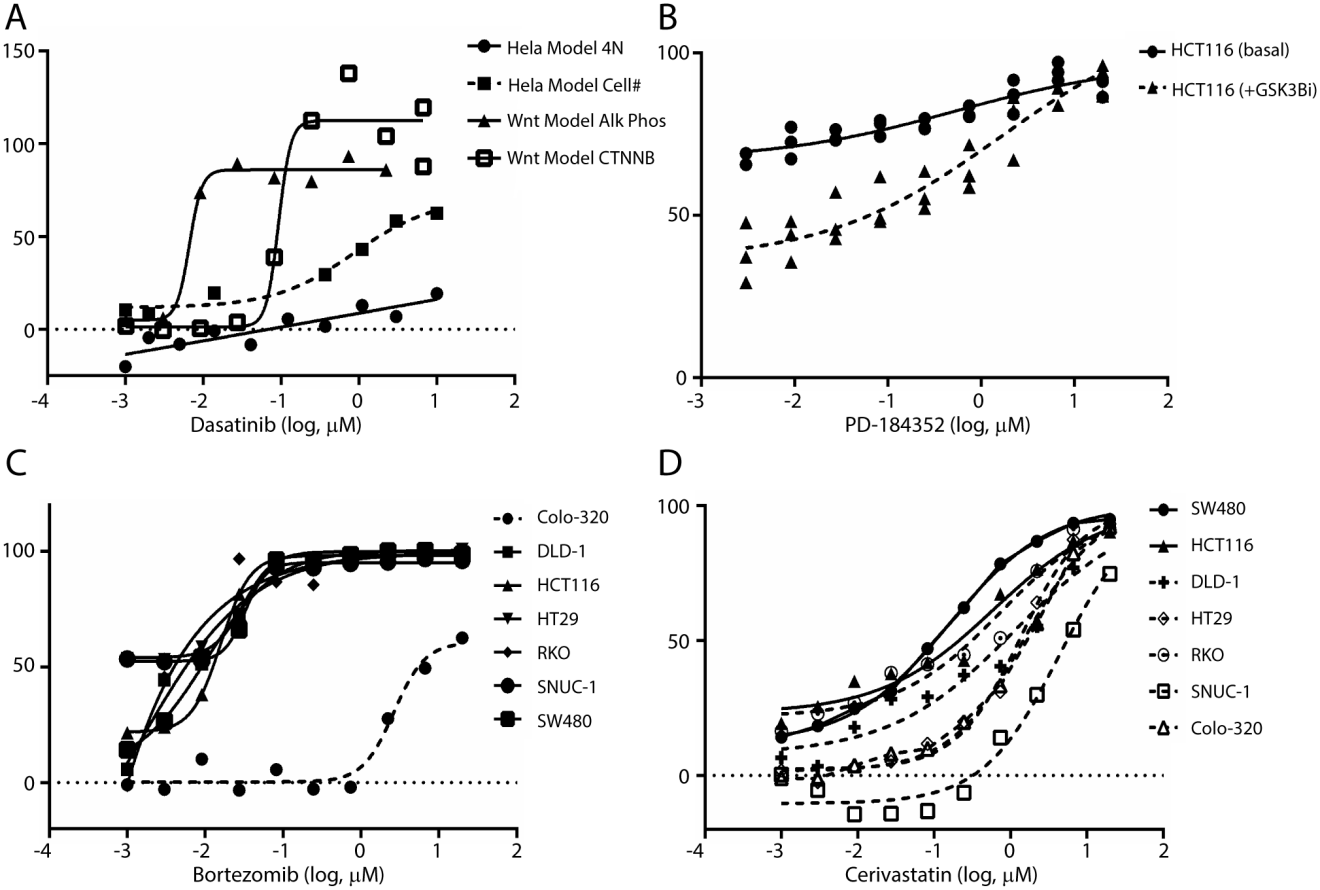
Activities for selected approved drugs versus specified assay modules. A response of 0% is equivalent to DMSO control, while a 100% response corresponds to the appropriate positive control for the assay (methods). A. The activity of the multikinase inhibitor dasatinib within the Wnt potentiation module. Included are the outcomes in a multilineage potential C2C12 cell line as judged by β-catenin translocation (□) and alkaline phosphatase activity (▲); selectivity of the Wnt response vs. cell cycle effects is assessed by comparison to % inhibition of cell count (■) and % stimulation of 4N DNA content (●) as determined via propidium iodide staining in HeLa cells. B. Inhibition of cellular ATP by the MEK inhibitor PD-184352 in HCT116 cells alone (●) or following pretreatment with a GSK3β inhibitor (▲). C. Inhibition of cellular ATP by the proteasome inhibitor bortezomib in the KRAS assay modules including Colo320 (-●-), DLD-1 (■), HCT116 (▲), HT-29 (▼), RKO (♦), SNU-C1 (●), and SW480 (■). D. Inhibition of cellular ATP by simvastatin, cerivastatin and fluvastatin in the KRAS assay modules including the KRAS-mutant lines SW480 (●), HCT116 (▲) and DLD-1 (+) vs. the KRAS wild type lines HT-29 (◊), RHO (○), SNU-C1 (□) and Colo320 (Δ).

The FDA approved multikinase inhibitor dasatanib provides insight into several assay modules. The broad scope of kinases targeted by this agent is often proposed as an explanation for its activity in multiple settings. Indeed, dasatinib was noted to possess modest activity in the KRAS assay modules (Table 2). A recent report highlighted that dasatinib sensitized mutant KRAS cells to cetuximab through down-regulation of broad-spectrum signaling pathways including MAPK and PI3K-AKT-mTOR [25]. Dasatinib also possesses potent stimulatory activity in the Wnt potentiation module (Table 2; Fig 2A). Interestingly, low-dose dasatinib has been noted to stimulate osteoblast formation while inhibiting osteoclast formation and resorption activity and promote trabecular bone formation in a murine model [26]. This report implicates the actions of dasatinib versus the platelet derived growth factor receptor-b (PDGFR- β), c-Src and c-Kit as a progenitor of the stimulated Wnt activity in hMSC-TERT and MG-63 lines. The NPC contains approved inhibitors of PDGFR-β, c-Src, and c-Kit, including Pazopanib, Vatalanib and Sorafenib. None of these agents, however, possessed appreciable activity in the Wnt potentiation module suggesting that the activity of dasatinib as a Wnt activator and a bone-modifying agent is the result of its activity versus targets as yet undefined and/or its cumulative polypharmacology.

Many of the aforementioned FDA approved kinase inhibitors possess activities in the angiogenesis assay module in concordance with known activity. The actions of other kinase inhibitors in the collection proved interesting. The MEK inhibitor PD-184352 inhibited endothelial tube formation (Table 2) consistent with the promotion of endothelial cell survival and sprouting by ERK-MAPK signaling [27]. Mitogen-activated protein kinase (MAPK) pathway signaling has been demonstrated to stimulate Wnt signaling in mutant KRAS colorectal cancers [28]. Indeed, activation of Wnt signaling *via* inhibition of GSK3β led to a resistance to PD-184352 and other MEK/Erk inhibitors subsequently tested in the HCT116 mutant KRAS line (Table 2; Fig 2B). These results validate the notion of cross-talk between MAPK and Wnt signaling pathways in mutant KRAS cancer lines. Confirmatory activities found in this data set were not limited to the kinase inhibitor class. The approved proteasome inhibitors bortezomib and carfilzomib were found to be highly active in the majority of cell lines in the KRAS assay module. The exception proved to be the Colo320 cell line (Table 2; Fig 2C). Interestingly, Colo320 has been demonstrated to be resistant to several therapeutic strategies and increased activity of the transcription factor nuclear factor E2-related factor 2 (Nrf2) which enhances expression and activity of key proteasome subunits [29]. Another intriguing result from the KRAS assay module was the activity of the statin class of drugs. Kang and coworkers have recently published that simvastatin resensitized KRAS mutant colorectal cancer to cetuximab through an apoptotic related mechanism involving mitigation of compensatory BRAF activity [30]. In our profiles, simvastatin, cerivastatin and fluvastatin each possessed single agent activity in KRAS mutant cell lines (SW480, DLD-1, HCT116). Cerivastatin and fluvastatin, but not simvastatin, exhibited inhibition of KRAS mutant cell lines SW480 and HCT116 with 5–10 fold lower potency vs. KRAS wild-type cell lines, Colo320 and SNU-C1; Table 2). Overall, cerivastatin possessed the strongest pan-activity and selectivity towards KRAS mutant cell lines (Fig 2D; Table 2). These data suggest a reexamination cetuximab+statin combination using cerivastatin in lieu of simvastatin. This outcome also highlights a key element of repurposing studies whereby drugs no longer in clinical use can find novel utility.

### Novel Activities

**Fig 3:**
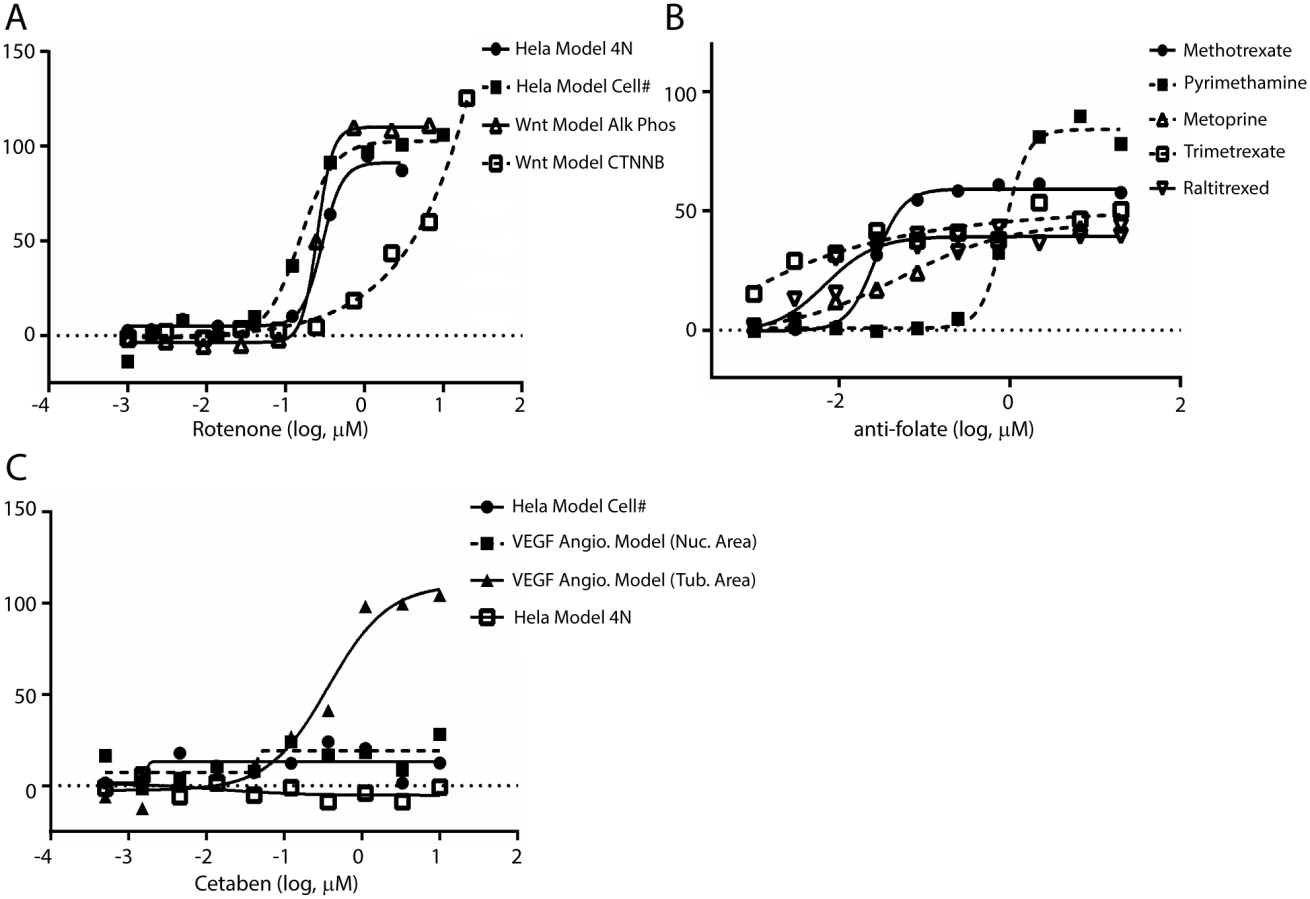
Novel activities for selected approved drugs versus specified assay modules. A. Potentiation of Wnt pathway signaling by rotenone. Included are the outcomes in a multilineage potential C2C12 cell line as judged by β-catenin translocation (□) and alkaline phosphatase activity (Δ); selectivity of the Wnt response vs. cell cycle effects is assessed by comparison to % inhibition of cell count (■) and % stimulation of 4N DNA content (●) as determined via propidium iodide staining in HeLa cells. B. Potentiation of Wnt pathway signaling by antifolates methotrexate (●), pyrimethamine (■), metoprine (Bw 197U; Δ), trimetrexate (□) and raltitrexed (∇) in the Wnt potentiation module. C. In vitro anti-angiogenic activity of cetaben. Inhibition of tube area in ECFC as measured via CD31 staining (▲) in the absence of ECFC/ADSC cytoxicity as assessed via Hoechst staining (■); selectivity of endothelial response vs. cell cycle effects is assessed by comparison to % inhibition of cell count (●) and % stimulation of 4N DNA content (□) as determined via propidium iodide staining in HeLa cells.

Novel activities were noted for several approved drugs (Table 2). Among these results was the noted activity of rotenone as an activator in the Wnt potentiation module (Fig 3A). Rotenone is a naturally occurring chromanone that is used as a broad spectrum pesticide/piscicide and a treatment for head lice in children. The reported mechanism of action for rotenone involves disruption of complex 1 within the mitochondrial electron transport chain. Recently, it was noted that low doses of rotenone lead to Parkinson disease (PD)-like symptoms leading researchers to conclude that low-doses of environmental toxicants that disrupt cellular respiration may be causal in the development of PD pathology over long exposure periods [31]. Interestingly, multiple reports associate disruption of Wnt signaling to altered cellular respiration and, specifically, mitochondrial physiology [32–36]. Rotenone and a structural congener deguelin are also highly utilized tool compounds based upon their well vetted ability to generate elevated ROS levels in cell culture settings [37]. The action of ROS (specifically H_2_O_2_) as signaling element is multifaceted and conflicting reports abound. Funato *et al*. have demonstrated nuclear β-catenin accumulation following low-level H_2_O_2_ exposure that was independent of the actions of Wnt [33]. Conversely, Almeida *et al*. have reported suppression of Wnt signaling in conditions of oxidative stress in osteoblasts and osteoblast precursors [32, 37]. Clearly the effects of ROS on cellular signaling events are complex and the results of our profile suggest that rotenone will be a useful tool in further shedding light on this domain of cellular biology.

The NPC collection contains several approved antifolates including pyrimethamine, metoprine, methotrexate, trimetrexate and raltitrexed. Each of these agents possessed an activation phenotype in the Wnt potentiation module (Table 2; Fig 3B). Antifolates target dihydrofolate reductase (DHFR) and lower the concentrations of reduced folate metabolites in the cell. The classical interpretation of their anticancer activities surrounds this limited pool of key metabolites for maintenance of normal cellular function and proliferation. Folate (and reduced folate metabolites) also plays a key role in epigenetic modulation as a key source of the single carbon utilized to methylate DNA with implications in developmental biology [38]. Antifolates have been entrenched in the pharmacopeia since aminopterin was described as an effective therapy in childhood leukemia in 1947. Further, methotrexate was approved for use in rheumatoid arthritis in 1988. Given the length of use of these drugs it is not surprising that there is a wealth of published information surrounding antifolates effect on cells. Several studies have provided information on the changes in gene expression signatures that occur following treatment of antifolates [39–41]. Included in these reports is evidence that β-catenin transcribed genes are altered and that Wnt associated developmental anomalies can be rescued through the actions of exogenously applied folate [39, 42, 43]. A ubiquitous cellular response to antifolates treatment is an upregulation in the expression of DHFR. Cleary and coworkers recently published the results of an RNAi screen (shRNA) designed to identify cellular targets that work in concert with a GSK3β inhibitor-stimulated β-catenin induced transcriptional program [44]. Among the top screening hit was DHFR which prompted an examination of methotrexate and GSK3β inhibition as a means to activate β-catenin. The positive outcome of these examinations highlighted the potential use of methotrexate and GSK3β inhibitors within inflammatory diseases. The outcome from our profile seems to support these data albeit without the need for concomitant inhibition of GSK3β.

The key goal of our Wnt potentiation module is to identify agents that may serve as novel medications for bone development and, specifically, osteoporosis. A recent examination of the U.S. Food and Drug Administration’s Adverse Event Reporting System (FAERS) was conducted to judge the utility of population pharmacology to predict the utility of drug combinations to overcome adverse events associated with single agent drug usage [45]. Among the supporting information was an analysis of femur fracture occurrence for patients on the bisphosphonate alendronate. The combinations analyzed included individuals taking both alendronate and methotrexate and indicated a reduction in this adverse event (reductions were also noted for aspirin, prednisone and furosemide among others) (S3 Table). Other studies suggest that methotrexate had no effect on non-vertebral fracture risk or even an associated increase [46, 47]. Given the favorable safety profile and the wealth of clinical data associated with the antifolate class of drugs, a more in-depth epidemiological examination into the potential role of these agents in osteoporosis is warranted.

Based on evidence highlighting the importance of angiogenesis for the growth and metastasis of solid tumors (see [48] for review), tumor neovascularization is considered one of the six fundamental hallmarks of cancer [49, 50]. VEGF and thrombospondin 1 were among the early endogenous factors found to stimulate and inhibit angiogenesis, respectively [48]. Today, net angiogenesis is known to be dependent on the counterbalance of at least two dozen endogenous activators and inhibitors originating from the extracellular matrix, tumor cells, and non-transformed cells of the tumor microenvironment [48–51]. To date, the majority of approved anti-angiogenesis drugs have been designed to either bind and neutralize VEGF directly or to inhibit the receptor tyrosine kinase which engages VEGF and related growth factors [52]. These drugs have shown clinical efficacy as monotherapies or in combination with chemotherapy through transient disease stabilization/tumor regression and increases in progression free survival (measured in months) but unfortunately do not increase overall patient survival [53–55].

Given that multiple mechanisms may contribute to the initial non-responsiveness observed in some patients and the inevitable acquired resistance of VEGF directed therapeutics, it is intriguing that cetaben, a drug candidate which failed phase 2 trials as an anti-arteriosclerotic agent due to lack of efficacy [56] demonstrates similar *in vitro* anti-angiogeneic activity as several receptor tyrosine kinase inhibitors (Table 2; Fig 3C) which have undergone clinical development and product launch. Although cetaben passes Lipinski’s Rule of 5 [57] most investigators would consider it “problematic” due to its highly aliphatic structure. Such compounds may act as detergents and show non-selective cytoxicity; however the phenotypic selectivity of cetaben in our assay panel suggests that the anti-angiogenic activity is not due to poor physical chemical properties leading to non-specific cell lysis, cytotoxicity, or ATP depletion (Table 2), observations which are consistent with the use of 100 μM cetaben to reversibly alter cell morphology [58].

The molecular mechanism by which cetaben inhibits endothelial cord formation is unknown.

Cetaben treatment lowers serum sterol and triglyceride levels in normal rats [59] and improves serum cholesterol/lipoprotein levels, blood flow, and vascular markers of diet induced atherosclerosis in non-human primates [60]. The NIH drug compound database annotates cetaben as a “cholesterol antagonist/inhibitor” however of the 16 molecules in this category and the 13 non-HMG Co-A reductase compounds classified to have “anti-cholesterol” activity, cetaben uniquely inhibited endothelial cord formation (Table 2). Six HMG Co-A reductase inhibitors were also tested in our 2D angiogenesis co-culture model with mixed results. Atorvastatin and pravastatin did not inhibit endothelial tube formation and were not cytoxic in the ECFC/ADSC co-culture system. In contrast cerivastatin, fluvastatin, simvastatin and lovastatin inhibited cord formation with IC_50_ values ranging from 0.1 to 3.3 μM but demonstrated overt cytoxicity in the assay system with IC_50_ values 2–3 fold larger than inhibition of endothelial cord formation. These results suggest that the observed effect of HMG Co-A reductase inhibitors are primarily due to a cytoxicity artefact in our 2D co-culture assay, an observation which contrasts with the results of Schulz *et al*. where HMG Co-A reductase inhibitors decreased sprouting in a 3D monoculture of microvasculature lymphatic endothelial cells that was rescued by addition of the product of HMG Co-A reductase, mevalonate and its metabolic derivatives farnesyl and geranyl pyrophosphate [61].

PubChem and ChEMBL extensively document cetaben’s *in vivo* activity on serum sterol and triglyceride levels in rodents. Inhibition of fatty acyl-CoA: cholesterol acyl transferase has been observed [59], but structure-activity studies of cetaben analogs suggest that cholesterol acyl transferase activity and the decrease in serum sterol and triglyceride levels observed *in vivo* do not follow a simple relationship [62, 63]. Cetaben does not inhibit the activity of KDR or 4 other protein kinases profiled in this study but decreased the incorporation of labeled acetate into fatty acids, triglycerides, and cholesterol esters but not cholesterol in HepG2 cells [64] suggesting a mechanism related to *de novo* fatty acid synthesis. This speculative mechanism for cetaben is consistent with the quantitative structure activity relationship between endothelial tube formation and acetyl-CoA carboxylase (ACC) activity [65]. In addition, endothelial tube formation but not nuclear count is inhibited by structurally diverse fatty acid synthetase (FASN) inhibitors. Specifically, heterocyclic FASN inhibitors described by Astra Zeneca [66–68] inhibited endothelial tube formation with IC_50_ values ranging from 19 nM to 1.8 μM and the aliphatic FASN inhibitor C75 [69] inhibited endothelial tube formation with an IC_50_ value of 3.7 μM. Taken together, these results suggest that cetaben suppresses angiogenesis through a mechanism which is distinct from the inhibition of receptor tyrosine kinases and which may involve *de novo* fatty acid synthesis. This proposed mechanism of action is consistent with studies demonstrating that the obesity drug orlistat, a natural product derivative initially developed as a lipase inhibitor [70] but subsequently shown to inhibit the thioesterase domain of FASN [71], inhibited angiogenesis in several models at high test dosages (>50–100 μM) [71, 72]. Reliable potency estimates could not be determined with the orlistat dosages used in this study (< 10 μM).

### Conclusions

Drug repositioning represents the swiftest way to bring new therapeutics to unmet medical needs. Phenotypic assays have a proven track-record for generating compounds with therapeutic potential. The screening of large libraries of approved drugs in well-engineered, high-throughput phenotypic assays provides a means to quickly assess new activities for the existing pharmacopeia. Here, we provide the primary outcomes for a large collection of approved drugs [The National Center for Advancing Translational Sciences (NCATS) pharmaceutical collection (NPC)] screening in the OIDD phenotypic modules offered by Lilly which include an osteoporosis model, two diabetes models, and two cancer models. The compilation of all related data is publically available online at www.ncats.nih.gov/expertise/preclinical/pd2 and via the PubChem Database (https://pubchem.ncbi.nlm.nih.gov/)(AID 1117321).

The OIDD phenotypic modules are designed to inform on common mechanisms that play a role in specific disease etiologies. For instance, the role of insulin secretion in diabetes as judged by a high-throughput amendable assay involving the rat insulinoma cell line INS-1E. The NPC collection of approved drugs includes many drugs approved for indications such as diabetes that are known to work through defined mechanisms like the stimulation of insulin secretion. The positive outcomes for known insulin secretagogues of the sulfonylurea and metaglinide classes present strong validation that these assays were, in fact, informing on relevant pharmacological outcomes. As a result, when novel actives are defined it offers insight into a drug’s full potential.

The activities of selected kinase inhibitors were intriguing. While several classes of kinase inhibitors are highly selective (MEK, covalent modifying kinase inhibitors) many possess a vast polypharmacology across the kinome target-scape. Approved drugs like dasatinib are now recognized to possess potent activity versus numerous kinases and it is likely that this ‘dirty’ pharmacology contributes to its phenotypic actions. Indeed, the screening of drugs with extensive polypharmacologies within phenotypic assays is an intriguing exercise in associating mechanism to phenotype. In our studies, dasatinib possessed activity in both the Wnt stimulation assay module and the KRAS assay module. Each of these phenotypic outcomes is in alignment with recent reports that demonstrate dasatinib’s potential to sensitize mutant KRAS cells to cetuximab and to stimulate osteoblast formation in a bone-forming model of osteoporosis [25, 26]. As the activity of dasatinib across the kinome is well documented, these results offer a means to selectively knock down the targets of dasatinib individually and in combination in hopes of finding the correct target(s) that manifest these activities.

One of the intriguing outcomes from this study was the pan-activity of the antifolates class of drugs within the Wnt potentiation module. Antifolates have been a mainstay of cancer and rheumatoid arthritis for decades and much is known about its phenotypic outcomes in patients on varying doses of these drugs. Further, given their longstanding role in the pharmacopeia, there is a significant amount of published data from the basic and translational research communities. As a result, the uniform activity of each approved antifolates in this assay module was a compelling outcome. Mechanistic insight into this finding was recently reported in the form of an RNAi screen that highlighted the role of DHFR as a protein target that works in concert with GSK3β inhibition to stimulate β-catenin activity. Further, an analysis of the U.S. Food and Drug Administration’s Adverse Event Reporting System (FAERS) suggested that the use of antifolates may limit the occurrence of femur fractures in patients taking the bisphosphonate alendronate. Our assay results and these studies suggest that a deeper investigation into the role of antifolates as Wnt stimulating agents is warranted.

The molecular mechanisms responsible for the failure of current anti-angiogenic therapies are unclear; multiple mechanisms contribute to intrinsic or acquired resistance [54]. When effective, current therapies lead to transient tumor regression and increased progression free survival (measured in months) but unfortunately not overall patient survival [53–55]. In addition, withdrawal of antiangiogenic therapies leads to enhanced tumor regrowth and metastasis; this rebound phenomena has been recently correlated with a shift in the metabolic state of the tumor tissue towards increased lipid metabolism as detected by transcriptional, proteomic, and metabolic endpoints [73]. Strikingly, pharmacological or genetic inhibition of FASN activity reduced the tumor rebound and metastasis in several tumor model systems [73].

The involvement of fatty acid synthesis with *in vivo* tumor rebound and metastasis following withdrawal of VEGF-directed anti-angiogenic therapies [73] and *in vitro* studies demonstrating that structurally distinct ACC inhibitors [65], structurally distinct FASN inhibitors (this work), and possibly cetaben inhibit angiogenesis by blocking *de novo* fatty acid biosynthesis is intriguing since fatty acid biosynthesis has been implicated in cancer pathogenesis on the basis of the glycolytic metabolism of tumor cells, the overexpression of FASN in various epithelial cancers, and the important role of lipogenic enzymes in tumor cell survival [74, 75]. Inhibition of *de novo* fatty acid biosynthesis may therefore provide multiple opportunities to modulate cancer pathogenesis, directly by interfering with tumor cell metabolism/survival, indirectly by inhibition of tumor neo vascularization, and by inhibiting tumor rebound and metastasis following withdrawal of current anti-angiogenic therapies. Further work investigating the potential synergism of agents directed towards *de novo* fatty acid biosynthesis and current VEGF-directed anti-angiogenic agents is warranted.

High-throughput methods involving phenotypic assay systems offer a means to generate insight into not only unbiased small molecule libraries but also libraries of approved drugs. To enable the research community methods to access to the PD2 assay modules and the NPC drug collection can be found at https://openinnovation.lilly.com/dd/ and http://www.ncats.nih.gov/research/tools/preclinical/npc/pharmaceutical-collection.html, respectively. Here, we report the results from screening a collection 2,426 approved drugs versus five phenotype informing assay modules including GLP-1 secretion (0.7% hit rate), Insulin secretion (0.6% hit rate), Wnt pathway potentiation (2.4% hit rate), KRAS synthetic lethality (1.4% hit rate) and an anti-angiogenesis model (3.7% hit rate). Importantly, all data is publically accessible at www.ncats.nih.gov/expertise/preclinical/pd2 and through the PubChem Database (https://pubchem.ncbi.nlm.nih.gov/)(AID 1117321). By making this large collection of data public we hope to enable the broader research community to explore hypothesis driven research aimed at the repositioning of individual drugs. Our experience in conducting phenotypic screens highlights the importance of considering newly-identified compound activities in the context of previous experience. Compounds exhibiting activity in one module that also show activity across several others are perhaps best deprioritized. The evaluation of phenotypic screen results on marketed drugs in the context of the data provided in this study should help identify drugs with the highest potential for repositioning as human therapeutics.

## Supporting Information

S1 Table Tabulation of assays used for profiling NPC compounds. (XLS)

S2 Table IC50 / EC50 in μM for 173 active compounds across 5 phenotypic assay modules. See legend for Table 2 in main manuscript
(XLS)

S3 Table Analysis of FDA adverse event (AE) reporting system data showing trends of reduced bone-related AEs for drugs co-administered with methotrexate. (XLS)

S4 Table Summary of concentration response assay results for 584 NPC compounds. All IC50/EC50 results are reported in μM; when a compound was tested multiple times in one assay, the result shown is summarized as described in methods.
(XLS)

S5 Table Summary of single concentration assay results for 2,509 NPC compounds. All IC50/EC50 results are reported as percent response in a signal window defined between a negative control (DMSO) and a positive control (see methods); for results obtained at different concentrations, or when a compound was tested multiple times in one assay, the result shown is the maximum of all test results for that compound.
(XLS)
